# Cognitive Strategies Regulate Fictive, but not Reward Prediction Error Signals in a Sequential Investment Task

**DOI:** 10.1002/hbm.22433

**Published:** 2013-12-31

**Authors:** Xiaosi Gu, Ulrich Kirk, Terry M Lohrenz, P Read Montague

**Affiliations:** 1 Wellcome Trust Centre for Neuroimaging, University College LondonLondon, United Kingdom; 2 Human Neuroimaging Laboratory, Virginia Tech Carilion Research InstituteRoanoke, Virginia; 3 Institute of Psychology, University of Southern DenmarkOdense, Denmark; 4 Department of Physics, Virginia Polytechnic Institute and State UniversityBlacksburg, Virginia

**Keywords:** decision-making, reward prediction errors, fictive learning, emotion regulation, reappraisal, insula, fMRI

## Abstract

Computational models of reward processing suggest that foregone or fictive outcomes serve as important information sources for learning and augment those generated by experienced rewards (e.g. reward prediction errors). An outstanding question is how these learning signals interact with top-down cognitive influences, such as cognitive reappraisal strategies. Using a sequential investment task and functional magnetic resonance imaging, we show that the reappraisal strategy selectively attenuates the influence of fictive, but not reward prediction error signals on investment behavior; such behavioral effect is accompanied by changes in neural activity and connectivity in the anterior insular cortex, a brain region thought to integrate subjective feelings with high-order cognition. Furthermore, individuals differ in the extent to which their behaviors are driven by fictive errors versus reward prediction errors, and the reappraisal strategy interacts with such individual differences; a finding also accompanied by distinct underlying neural mechanisms. These findings suggest that the variable interaction of cognitive strategies with two important classes of computational learning signals (fictive, reward prediction error) represent one contributing substrate for the variable capacity of individuals to control their behavior based on foregone rewards. These findings also expose important possibilities for understanding the lack of control in addiction based on possibly foregone rewarding outcomes. *Hum Brain Mapp 35:3738–3749, 2014*.

## INTRODUCTION

Recent computational models and experimental probes support the notion of multiple learning mechanisms in healthy individuals [Chiu et al., 2008; Daw et al., 2011; Glascher et al., 2010; Lohrenz et al., 2007; Montague et al., 2004,^2006^; Pagnoni et al., 2002; Simon and Daw, 2011]. Reward prediction errors derived from ongoing differences between expected and actually experienced rewards (temporal difference (TD) errors) have a significant impact on choice behavior [Montague et al., 2004,^2006^; Schultz et al., 1997]. However, these signals do not fully capture the complexity of decision-making processes. Recent evidence demonstrates that learning can also be driven by fictive errors derived from foregone outcomes (“what might have happened”) [Chiu et al., 2008; Hayden et al., 2009; Lohrenz et al., 2007]. One central physical substrate supporting these mechanisms is dopaminergic signaling in the brain [Niv et al., 2005; Rangel et al., 2008]. In healthy individuals, both fictive [Lohrenz et al., 2007] and reward prediction errors [Montague et al., 2002] activate the striatum [Chiu et al., 2008; Montague et al., 2002], a dopaminoceptive structure that is commonly implicated in decision-making tasks and works closely with a network of brain regions such as the anterior insular cortex (AIC), orbitofrontal cortex (OFC), and the amygdala [Hsu et al., 2005; Li et al., 2011; Seymour et al., 2004].

An outstanding question is how these computational learning signals interact with top-down cognitive influences [Dayan et al., 2000; Montague et al., 2004]. In people with compromised top-down control (e.g. addicted individuals), fictive errors are computed in the brain, but fail to emerge as signals to guide choice behavior [Chiu et al., 2008], suggesting that fictive learning signals might interact with top-down cognitive input. On the other hand, cognitive strategies used to regulate emotions such as reappraisal have been shown to modulate neural activity related to reward anticipation [Delgado et al., 2008; Staudinger et al., 2011], loss aversion [Sokol-Hessner et al., 2013; Sokol-Hessner et al., 2009], and risky choices [Martin and Delgado, 2011] during decision-making. With the consideration of individual differences in learning and decision-making [Chiu et al., 2008; Daw et al., 2011; Glascher et al., 2010; Lohrenz et al., 2007; Montague et al., 2004,^2006^; Pagnoni et al., 2002; Simon and Daw, 2011], it is therefore important to investigate the interplay between emotional regulation strategies and computational learning signals in individual decision-makers, which might open a window into intervention and treatment of psychiatric conditions with abnormal decision-making patterns.

**Figure 1:**
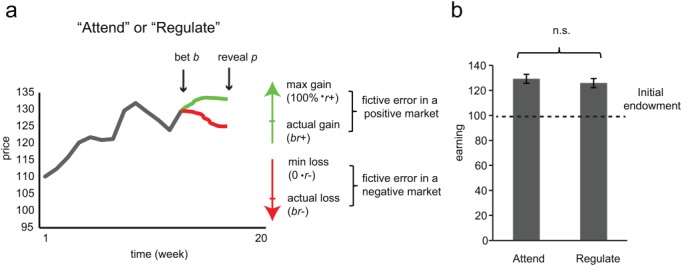
a) Experimental paradigm. Participants made investment choices under two task conditions: a cognitive reappraisal task “Regulate,” and a control task “Attend.” At each time point, the subject makes an investment decision *b* (0–100% of current portfolio). The market price *p* is then revealed with two possible directions of outcomes: increase or decrease. In a positive market where market return *r^+^* > 0, the best fictive outcome would be having invested 100%, therefore the fictive error in a positive market *f^+^* =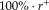 – *br^+^*. Conversely, when the market return is negative (*r*^–^ < 0), the best fictive outcome would be having invested nothing, therefore the fictive error in a negative market *f^−^* = 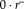 – *br^−^*. b) There was no significant difference in overall earning between Attend and Regulate strategies (*N* = 63). Each subject was endowed with 100 money units to invest. n.s., not significant. Error bars represent standard error.

In the current study, we investigated the impact of cognitive influences implemented through an emotion regulation strategy on fictive and reward prediction error signals in healthy adults. We employed a sequential investment task (Fig.1a; modified from [Chiu et al., 2008; Lohrenz et al., 2007]) and functional magnetic resonance imaging (fMRI), together with a cognitive reappraisal strategy, to frame subjects on the overall earnings based on their decisions (“Regulate”; see Materials and Methods and Supporting Information), in comparison to a control strategy focused on each local decision (“Attend”). Similar cognitive strategies have been proven successful in regulating emotions in both clinical [Kober et al., 2010; Volkow et al., 2010] and nonclinical settings [Delgado et al., 2008; Gross, 1998; Ochsner et al., 2012; Wager et al., 2008]. In the current sequential investment task, the Regulate strategy could modulate (1) neither fictive nor reward prediction errors, (2) both fictive and reward prediction errors, (3) fictive signals only, (4) reward prediction errors only. Our working hypothesis was that the Regulate scenario would be associated with a diminished impact of the fictive error only because of its status as a learning signal generated by foregone choices (“what might have been” had decisions been different) and its reported vulnerability to changes in psychophysiological states [Chiu et al., 2008]. Under this hypothesis, we should expect an attenuation of the weight of fictive errors on a subject's next bet and this attenuation should be accompanied by reduced neural responses to fictive errors but not reward prediction errors (the “experienced” errors in our description above). We also explored individual differences in fictive and reward prediction error learning signals.

## MATERIAL AND METHODS

### Participants

Seventy healthy adults were recruited from community populations in Virginia (*N* = 63) and London (*N* = 7). Seven participants recruited in Virginia were excluded due to excessive head motion (>4 mm), yielding a final sample of 63 healthy adults (age mean ± standard deviation (SD): 32 ± 13 years; 34 females; 56 from Virginia and 7 from London) with normal or adjusted to normal vision, no contraindication to MRI, and reported no previous or current psychiatric or neurological conditions. Subjects were informed of the study requirements and provided written consent prior to participation. The study was approved by the Institutional Review Board of Virginia Tech and the University College London ethics committee.

### Stimulus and Procedure

Participants performed a sequential investment task (Fig. 1a) under two task conditions: a cognitive reappraisal task “Regulate,” and a control task “Attend” (see Supporting Information for a full description). There were ten “Regulate” markets and ten “Attend” markets, presented in a randomized order for each participant. The Regulate scenario focused the subjects on their entire sequence of choices and the overall performance (e.g. “… Remind yourself that you are making many of these similar decisions. Do not keep a running total—simply approach these investment decisions keeping in mind their context”); it also requires the subjects to take the perspective of a trader (e.g. “…You take risks with money every day, for a living. All that matters is that you come out on top in the end - a loss or gain here or there will not matter in terms of your overall portfolio. In other words, you win some and you lose some”). Such strategies have been shown to reduce loss aversion in laboratory settings [Sokol-Hessner et al., 2009,^2013^] and relate to trader performance in real-life investment scenarios [Fenton-O'Creevy et al., 2011]. In contrast, the Attend strategy (see [Sokol-Hessner et al., 2009,^2013^]) clearly tells the subject that every decision counts and that she should take the perspective of her own.

We used twenty historic stock markets, similar to the ones used in previous studies (see [Chiu et al., 2008; Lohrenz et al., 2007]). There were 10 “Regulate” markets and 10 “Attend” markets, presented in a randomized order for each participant. Each market was considered a task block; there were 20 events (i.e. twenty investment decisions) in each market, yielding a total of 400 trials (200 trials for “Attend” and 200 trials for “Regulate”). The average task duration was approximately 35 min (ranging from 20 to 54 min; mean ± SD: 2,077 ± 460 s). Detailed task instructions were given to the subjects upon their arrival (outside of the scanner), and were repeated right before the actual scanning started (inside the scanner). During scanning, subjects saw a screen with the word “Attend” or “Regulate” which indicated the task for each market/block at the beginning of each market.

Participants were informed that they would have $100 US Dollars (Virginia) or £100 British Pounds (London) as their initial portfolios (i.e. total amount of money they have at a given time point) at the beginning of the experiment, and were informed that their final payment would be scaled according to their score in the experiment. No payment was given to the subjects before the experiment. At each time point *t*, the subject used a two-button box to move a slide bar to make an investment decision *b_t_* (0–100% of current portfolio) without a time constraint. Their mean response times ranged from 600 ms to 1,702 ms (mean ± SD: 1,075 ± 261 ms); 750 ms after they submitted their choices, the market price *p_t_* was revealed and the fractional market price change and subjects' portfolio were updated. Market information for all previous segments then remained on the screen. The slide bar then changed from gray to red after another 750 ms, and subjects started to make investment decisions for the next market segment. There are two possible directions of outcomes: increase or decrease. Trials with increased or decreased market price compared with the previous time point were considered positive and negative markets, respectively. The market return *r_t_* equals (*p_t_* − *p_t_*_ − 1_)/*p_t_*_ − 1_ and the gain is defined as *g_t_* = 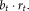 In a positive market where *p_t_* − *p_t_*_ − 1_ > 0, *r_t_^+^* <0, and positive gain *g_t_^+^ =*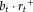, the best fictive outcome would be having invested 100%, therefore, the positive fictive error *f^+^* = 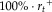 –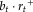
(or *f^+^* = *r_t_^+^* – *g_t_^+^*). Conversely, when the market return is negative (*p_t_* − *p_t_*_ − 1_ < 0, *r_t_^−^* < 0, and negative gain *g_t_*^−^ *=*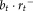), the best fictive outcome would be having invested nothing; therefore, the fictive error in a negative market *f*^−^ = 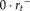 − 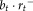 (or *f*^−^ = − *g_t_*^−^). We will focus our main analyses on *f^+^* based on previous research suggesting that in healthy participants, investment behavior is mostly driven by *f^+^*, but not *f*^−^ [Chiu et al., 2008; Lohrenz et al., 2007].

### Behavioral Data Analysis

We regressed the subject's next bet (*b_t + 1_*) against previous bet (*b_t_*), positive and negative market return (*r_t_^+^* and *r_t_*^−^ respectively), and the interaction terms (*b_t_ r_t_^+^* and *b_t_ r_t_*^−^) simultaneously cross task conditions by coding Attend and Regulate as two indicator variables (*regstats* function in MATLAB, R2012a, The MathWorks, Inc, Natick, MA): 
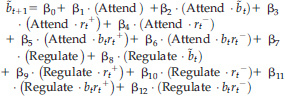


Here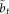 is the within-subject *z*-normalized bet. Because the data were pooled across subjects, and because we are primarily interested in the within-subject trial-to-trial fluctuations rather than the subject's general level of bet, we *z*-normalized the bets within subject so that they are comparable among subjects. Differences between regression coefficients of two task conditions were tested by performing linear hypothesis tests of the regression coefficients (F tests) using *linhyptest* in MATLAB.

We also assessed the influence of TD errors on subjects' next investment choice with a multiple regression model with the previous bet (*b_t_*) and TD as regressors: 
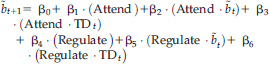
where TD*_t =_*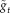 − 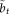, and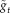 is within-subject *z*-normalized gain/loss. In other words, TD represents the difference between the actual gain at time *t* and the expected gain at that time, which corresponds to the bet. This definition of TD requires the gain to be comparable in scale to the bet; therefore, we also *z*-transformed the gain term, considering we already *z*-scored the bet term. Statistical significance was determined at *P* < 0.05 two-tailed.

We also explored individual differences in the extent to which fictive error signals influence investment decisions both behaviorally and neurally in a post hoc analysis. As established previously, the fictive error *f^+^* is constructed as the difference between the maximum fictive gain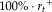 and the actual gain
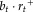


 Therefore, for *f ^+^* to exert an impact on one's behavior, two criteria must be met: (1) the influence of *r_t_^+^* on the next bet must be positive, and (2) the influence of *b_t_ r_t_^+^* on the next bet must be negative. Considering that the impact of *r_t_^+^* on the next bet is positive in most individuals (3 out of 63 individuals showed negative beta weights of *r_t_^+^* and were excluded from this analysis), we identified different learning strategies by examining the direction of the impact of *b_t_ r_t_^+^* on the next bet for each individual. We identified two groups of subjects groups as fictive learners (*f*-learners, *N* = 31) and temporal difference learners (TD-learners, *N* = 29; Fig. 4) respectively: *f*-learners' next bets were negatively driven by *b_t_ r_t_^+^*, and therefore significantly influenced by *f^+^* while TD-learners' behavior were positively driven by *b_t_ r_t_^+^*(see Results for details).

### Image Acquisition and Preprocessing

The anatomical and functional imaging was conducted on two identical 3.0 Tesla Siemens Trio scanners in Virginia and one 3.0 Tesla Siemens Trio scanner in London. Scanner and country were coded as nuisance variables at the second level fMRI analysis. High-resolution T1-weighted scans (1.0 mm × 1.0 mm × 1.0 mm) were acquired using an MP-RAGE sequence. Functional images were acquired using echo-planar imaging (EPI), and angled 30° with respect to the anterior-posterior commissural line. The detailed settings for the functional imaging were: repetition time (TR) = 2,000 ms; echo time (TE) = 25 ms; flip angle = 90°; 37 slices; voxel size: 3.4 mm × 3.4 mm × 4.0 mm. The functional scans were adjusted for slice timing, realigned to the first volume, coregistered to the T1 image, normalized to a standard template (MNI, Montreal Neurological Institute), and spatially smoothed with an 8 × 8 × 8 mm full-width-at-half-maximum (FWHM) Gaussian kernel.

### fMRI Data Analysis

Event-related analyses of the fMRI data were conducted using statistical parametric mapping (SPM8; Wellcome Department of Imaging Neuroscience, London, UK). General linear modeling (GLM) [Friston et al., 1995] was conducted for the functional scans from each participant by modeling the observed event-related blood-oxygen-level dependent (BOLD) signals and regressors to identify the relationship between the task events and the hemodynamic response. Regressors of 0 s duration related to visual and motor events were created by convolving a train of delta functions representing the sequence of individual events with the default SPM basis function, which consists of a synthetic hemodynamic response function (HRF) composed of two gamma functions [Friston et al., 1998]. The regressors include: market type screen; initial market history screen; key press; Attend: reveal of market price of first round; Attend: reveal of rounds 2 to 19; Attend: reveal of market price of last round; Regulate: reveal of market price of first round; Regulate: reveal of rounds 2 to 19; Regulate: reveal of market price of last round. Six parameters generated during motion correction were entered as covariates. TD and fictive errors (*f^+^, f^−^*) were entered as parametric regressors at the onsets of revealing the market prices. Although the lack of time constraints in the decision period and the lack of jitter may compromise the efficiency of this task for fMRI, using TD and fictive errors derived from model-based approaches as parametric modulation could potentially increase the efficiency compared with contrast-based approaches. Linear contrasts of the parameter estimates were made to identify the effects of temporal difference errors and fictive errors (TD*, f^+^, f^−^*) under Attend and Regulate, and their differences, for each participant. These images from all participants were then entered into a second-level group analysis conducted with a random-effects statistical model. One-sample *t*-tests were conducted for effects common to all participants and two-sample *t*-tests for group comparisons between the two types of learners. Significant activations related to the effects of TD errors under Attend are Regulate were identified at *P* < 0.05 level corrected for family-wise errors (FWE). All other activations were identified with a height threshold of *P* value exceeding 0.005 uncorrected in conjunction with an extend threshold of 10 voxels (resampled as 2 × 2 × 2 mm) to maintain a balance between Type I and Type II errors (Lieberman and Cunningham, 2009]. Unbiased regions of interest (ROIs) were created using the MarsBaR toolbox (http://marsbar.sourceforge.net/) based on the main effect of fictive error averaged cross both task conditions. These ROIs include left anterior insular cortex (centered at [−34, 16, −12]), right lateral orbitofrontal coertex (LOFC; centered at [44, 22, −8]), right medial orbitofrontal cortex (MOFC; centered at [−4, 32, −16]), and left striatum (centered at [−16, −4, 14]), with 4 mm radius. Individual subject's parameter estimates were then extracted from each ROI for each task condition.

### Psychophysiological Interaction (PPI) Analysis

We conducted PPI analysis [Friston et al., 1997; Gitelman et al., 2003] to explore such the functional connectivity between AIC and other regions under the modulation of the reappraisal strategy. The bilinear term in PPI represents the interaction between physiological activity and a psychological context input, which modulates the connectivity between the seed voxel of interest (VOI) and other brain regions, and has a directional character [Stephan et al., 2004]. The time series data of the first eigenvariate of the left AIC seed VOI derived from the ROI analysis were temporally filtered and mean corrected as in conventional SPM analysis. Bayesian estimation was used to deconvolve the time series of the BOLD signal to generate the time series of the neuronal signal for the VOI. The time series of the neuronal signal for responses to the events were created, resulting in one vector (the PPI regressor) representing the interaction between the reappraisal strategy and the AIC VOI (the psychophysiological interaction variable), a second vector (the P regressor) representing the contrast of fictive error-related Attend versus Regulate difference (the psychological variable), and a third vector (the Y regressor) representing the AIC VOI time course (the physiological variable). These regressors were forward-convolved with the canonical HRF, and then entered into the regression model along with vectors for other events. Model estimation was performed and the resulting SPM showed areas with significant differential connectivity to the VOIs due to context manipulations. The PPI analysis was carried out for each subject and the resulting images of contrast estimates were entered into a random effects group analysis. The statistical significance was set at a height threshold of *P* value exceeding 0.005 uncorrected in conjunction with an extend threshold of 10 voxels.

## RESULTS

### Behavioral Modulatory Effect on Fictive Errors

The reappraisal strategy (“Regulate”) did not significantly change overall earning compared with the control task (“Attend”) (*P* > 0.05; Fig. 1b). Subjects' raw investment levels were higher in the Regulate condition (mean ± SD: 45 ± 15%) compared with the Attend condition (mean ± SD: 42 ± 16%; paired *t*-test *P* = 0.014), consistent with previous finding on reduced loss/risk aversion under a similar reappraisal strategy [Sokol-Hessner et al., 2009]. The normalized bets were not significantly different between conditions (*P* > 0.9). In the Attend condition, the previous bet *b_t_*, positive and negative market return *r_t_^+^* and *r_t_*^−^, and the positive interaction term *b_t_ r_t_^+^* significantly predicted the next bet *b_t_*_ + 1_ (all *P*s < 0.001; Table1). These results are consistent with previous results that fictive gain signals guided behavior in healthy adults [Chiu et al., 2008; Lohrenz et al., 2007].

**Table 1:** Behavioral regression results

	Regressor	Coefficient	SE	*t* value	*P*
Model 1	Attend	1.98	7.66	−0.26	0.79
	Attend · b	0.62	0.01	71.30	<0.0001
	Attend · r+	4.93	0.29	17.29	<0.0001
	Attend · r−	−3.29	0.28	−11.94	<0.0001
	Attend · br+	−2.96	0.58	−5.07	<0.0001
	Attend · br−	−0.15	0.55	−0.27	0.78
	Regulate	1.98	7.66	0.26	0.80
	Regulate · b	0.63	0.01	73.58	<0.0001
	Regulate · r+	4.23	0.31	13.83	<0.0001
	Regulate · r−	−3.64	0.31	−11.93	<0.0001
	Regulate · br+	−0.69	0.60	−1.14	0.25
	Regulate · br−	−1.42	0.60	−2.38	0.02
Model 2	Attend	2.08	7.76e+10	0.27	0.79
	Attend · b	0.81	0.01	79.87	0.01
	Attend · TD	0.23	0.008	30.12	0.01
	Regulate	2.08	7.76e+10	0.27	0.79
	Regulate · b	0.86	0.01	84.01	<0.0001
	Regulate · TD	0.25	0.008	32.65	<0.0001

Model 1: multiple regression model with next bet as dependent variable and the following regressors: *b*, investment decision; *r+*, positive market return; *r−*, negative market return; *br+*, actual gain in positive markets; *br−*, actual loss in negative markets. Model 2: multiple regression model with next bet as dependent variable and the following regressors: *b*; TD, temporal difference error. SE, standard error.

**Figure 2:**
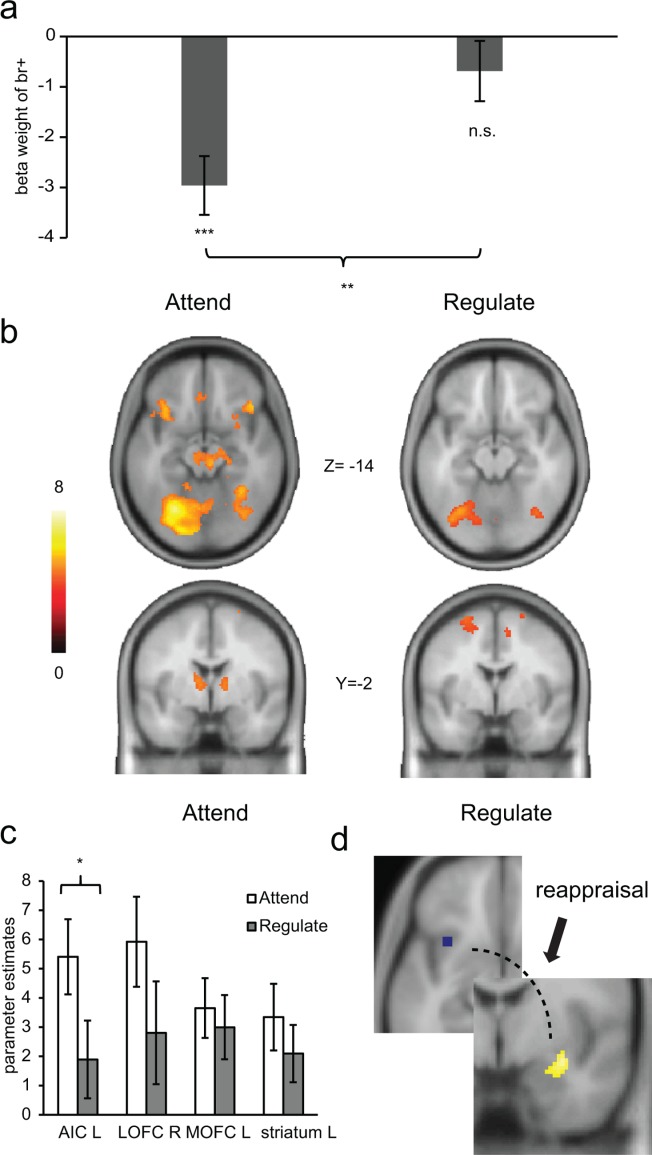
Behavioral and neural modulatory effects on fictive errors (*N* = 63). a) The reappraisal strategy significantly attenuated the beta weights of *br^+^* on the next bet. b) Fictive errors *f ^+^*activate the striatum, anterior insular cortex (AIC), lateral (LOFC) and medial orbitofrontal (MOFC) cortices, and midbrain nuclei, in the Attend, but not in the Regulate condition (*P* < 0.005 uncorrected). c) Region of interest analysis suggests that most robust *f^+^*-related task difference is in left AIC. d) Functional connectivity analysis suggests that AIC-amygdala connectivity is decreased by the reappraisal strategy (*P* < 0.005 uncorrected). Blue dot indicate the seed voxels in the left AIC utilized in the PPI analysis. Decreased connectivity in the right amygdala is displayed on coronal slice. ***P < 0.001, ** *P* < 0.01, *P < 0.05; n.s., not significant. L, left. *br^+^*: interaction term of bet (*b*) and positive market return (*r*^+^). Error bars represent standard error.

Importantly, when participants were asked to perform the Regulate strategy, *b_t_ r_t_^+^* no longer significantly predicted the next bet *b_t_*_ + 1_ (*P* > 0.05; Fig. 2a), while the regression coefficients of *b_t_*, *r_t_^+^*, *r_t_^−^* (all *P*s < 0.001) and the negative interaction term *b_t_ r_t_*^−^ (*P* < 0.05), were significant (Table1). We then tested the significance of differences between these regression coefficients under Attend and Regulate (Table2). Only the regression coefficients for *b_t_ r_t_^+^* were significantly different between Attend and Regulate (*P* = 0.007; Fig. 2a). Direct comparison between Attend and Regulate using individual betas showed a similar pattern: only the betas of the *b_t_ r_t_^+^* term were attenuated under Regulate (paired-*t* test *P* = 0.06; all other *P*s > 0.1). Fictive errors were correlated with TD errors under the Attend (*r* = 0.35, *P* < 0.01), but not Regulate condition (*r* = 0.11, *P* > 0.3); the difference between these two correlation coefficients was not significant (*P* > 0.1). There was no significant effect of site for any of these behavioral coefficients (all *P*s > 0.5). These results suggest the reappraisal strategy significantly attenuated the influence of fictive errors on investment behavior.

**Table 2:** Linear contrasts of regression coefficients

	Contrast	*F* value	*P*
Model 1	Attend · b vs. Regulate · b	0.99	0.32
	Attend · r+ vs. Regulate · r+	2.82	0.09
	Attend · r− vs. Regulate · r−	0.70	0.40
	Attend · br+ vs. Regulate · br+	7.37	0.007
	Attend · br− vs. Regulate · br−	2.44	0.12
Model 2	Attend · b vs. Regulate · b	8.50	0.004
	Attend · TD vs. Regulate · TD	3.67	0.06

Model 1: multiple regression model with next bet as dependent variable and the following regressors: *b*, investment decision; *r+*, positive market return; *r−*, negative market return; *br+*, actual gain in positive markets; *br−*, actual loss in negative markets.

Model 2: multiple regression model with next bet as dependent variable and the following regressors: *b*; TD, temporal difference error. SE, standard error.

### Neural Modulatory Effect on Fictive Errors

In parallel with the behavioral findings, we identified robust fictive error *f^+^* related activation in the striatum, AIC, lateral, and medial orbitofrontal cortices (LOFC and MOFC), and midbrain nuclei (substantia nigra and red nucleus), in the Attend condition (Fig. 2b and Supporting Information Table S1; *P* < 0.005 uncorrected and k > 10). Importantly, these activations were attenuated in the Regulate condition (Fig. 2b and Supporting Information Table S2). A direct comparison between Attend and Regulate showed significant attenuation in *f^+^* related activity in left AIC (extending into putamen) and inferior frontal gyrus (IFG) including LOFC (Supporting Information Table S3). The reverse contrast did not yield any significant activation, suggesting that Regulate did not enhance *f^+^* related activity compared with Attend. There was no significant effect of site in the striatum, insula, or IFG/OFC.

We further conducted unbiased ROI analysis based on seeds selected from the main effect of fictive error averaged across both task conditions (Fig. 2c and Supporting Information Table S4), including AIC (centered at [−34, 16, −12]), LOFC (centered at [44, 22, −8]), MOFC (centered at [−4, 32, −16]), and striatum (centered at [−16, −4, 14]). Paired *t*-test suggests that left AIC activity showed a significant reduction under Regulate, compared with Attend (*P* < 0.05). Although striatum and other ROIs showed a similar pattern, the differences between Regulate and Attend did not reach statistical significance (all *P*s > 0.05). These results suggest that the attenuation in the weight of fictive errors on behavior under the Regulate condition is predominantly accompanied by reduced activation in AIC.

We then explored changes in functional connectivity parameters using the same AIC ROI as our seed region (centered at [−34, 16, −12]). Relative to Attend, the Regulate strategy significantly decreased the functional connectivity between AIC and the amygdala (Fig. 2d and Supporting Information Table S4). Regulate did not enhance the functional connectivity between AIC and other regions at the same threshold (*P* < 0.005 uncorrected, *k* > 10). The amygdala has been implicated in a wide range of processes involving aversive emotions [Delgado et al., 2008; LaBar et al., 1998; Sokol-Hessner et al., 2013]. Therefore, these results together suggest that negative feelings associated with fictive error signals were likely to be reduced in the Regulate condition.

### Behavioral and Neural Modulatory Effects on Reward Prediction Errors

**Figure 3:**
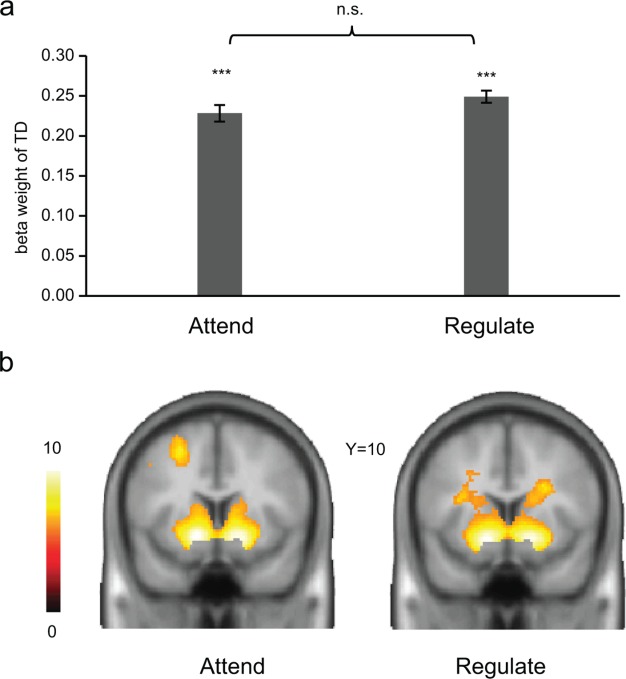
Behavioral and neural modulatory effects on reward prediction errors (*N* = 63). a) The reappraisal strategy did not change the beta weights of temporal difference (TD) errors on the next bet. b) TD errors activate the striatum and other reward-related brain regions in both Attend and Regulate conditions (*P* < 0.05 corrected for family-wise error). ***P < 0.001; n.s.: not significant. Error bars represent standard error.

We then examined the effects of reappraisal on reward prediction errors represented by temporal difference (TD*_t_*), where TD*_t_* is computed as the ongoing difference between the *z*-scored gained reward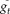 and the expected reward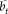 (i.e. TD*_t_ =*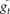 -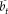, where *g_t_*=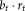). A behavioral regression model was carried out with normalized bet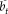 and TD*_t_* as predictors against the next bet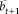. TD*_t_* significantly predicted the next bet under both Attend and Regulate conditions (both *P*s <0.001; Table1). The beta coefficients of TD*_t_* did not differ between Attend and Regulate (*P* > 0.05; Fig. 3a and Table2), suggesting that the reappraisal strategy did not significantly modulate the influence of reward prediction errors on choice behavior. Direct comparison between Attend and Regulate using individual betas showed a similar pattern: the betas of the TD*_t_* term were not significantly different (paired *t*-test *P* > 0.1). There was no significant effect of site for these behavioral coefficients (all *P*s > 0.5).

**Figure 4:**
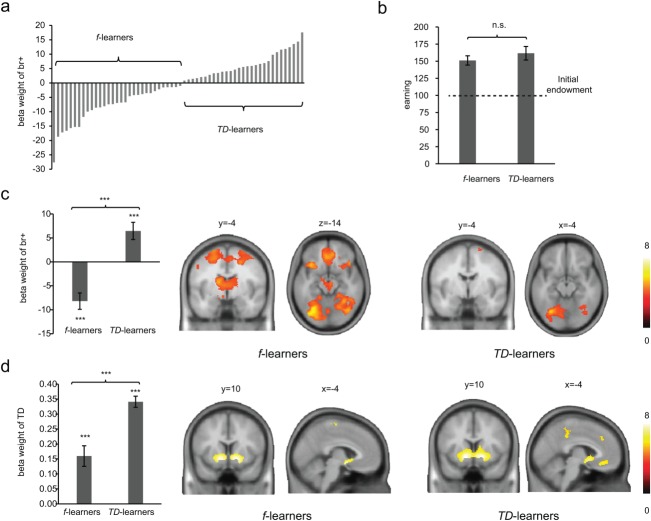
Individual differences in fictive and reward prediction learning. a) Fictive learners (*f*-learners, *N* = 31) show negative beta coefficients of *br^+^* (interaction term of bet *b* and positive market return *r*^+^), while temporal difference learners (TD-learners, *N* = 29) show positive beta weights. b) There is no difference in overall earnings between *f*-learners and TD-learners. c) In parallel with the behavioral difference between beta weights of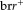 of the two groups (left), *f*-learners, but not TD-learners, show fictive error *f ^+^*-related activation in the insula, striatum, orbitofrontal cortex, and other related brain regions (*P* < 0.005 uncorrected). d) TD-learners have greater beta coefficients of TD compared with *f*-learners, accompanied by TD-related brain activations in orbitofrontal cortex (*P* < 0.05 corrected for family wise error). ***P < 0.001. n.s.: not significant, Error bars represent standard error.

Neurally, both Attend and Regulate yielded significant activation in the striatum, OFC, and other related brain regions (Fig. 3b and Supporting Information Tables S6 and S7; *P* < 0.05 corrected for family-wise errors and *k* > 5). A direct comparison between conditions did not reveal significant differences in TD-related activation in the AIC, striatum or other regions of interest (*P* < 0.005 uncorrected and *k* > 10; Supporting Information Table S8), although middle temporal/hippocampal activation was increased under Regulate. Direct comparison of individual parameter estimates of ROIs (AIC, LOFC, MOFC, and striatum) confirmed the GLM results: there was no significant difference in TD-related neural activity between Attend and Regulate in our regions of interest (all *P*s > 0.2). There was no significant effect of site in the striatum, insula, or IFG/OFC. Taken together, these behavioral and neural findings suggest that TD errors were not significantly modulated by the reappraisal strategy in the current investment task.

### Individual Differences: f-Learners and TD-Learners

In a post hoc exploratory analysis on individual differences, we identified two groups of subjects groups as fictive learners (*f*-learners, *N* = 31) and temporal difference learners (TD-learners, *N* = 29; Fig. 4) respectively: *f*-learners' next bets were negatively driven by *b_t_ r_t_^+^*, and therefore significantly influenced by *f^+^* while TD-learners' behavior were positively driven by *b_t_ r_t_^+^* (both *P*s < 0.001, two-sample *t*-test *P* < 0.001; Fig. 4c). Although both groups' behaviors were significantly driven by TD (both *Ps* < 0.001), TD-learners were more dependent on TD errors compared with *f*-learners (Fig. 4d; two-sample *t*-tests *P*s < 0.001). The overall earning did not differ between these two types of learners (Fig. 4b).

In line with their behavioral differences, *f*-learners and TD-learners also showed different patterns of brain activations related to fictive and reward prediction errors (Fig. 4c,d, and Supporting Information Tables S9–S12). *f-*learners showed robust *f^+^*-related responses in the striatum, OFC and AIC, while these neural responses were absent in TD-learners at the same threshold (Fig. 4c; Supporting Information Table S9). Direct comparison between groups confirmed that *f*-leaners showed greater activation in the AIC and OFC than TD-learners (Supporting Information Table S10). On the other hand, while both *f*-learners and TD-learners showed robust TD-related activation in the striatum and OFC, TD-learners showed significantly stronger TD*-*related activation than *f*-learners in the OFC (Fig. 4d; Supporting Information Tables S11 and S12).

**Figure 5:**
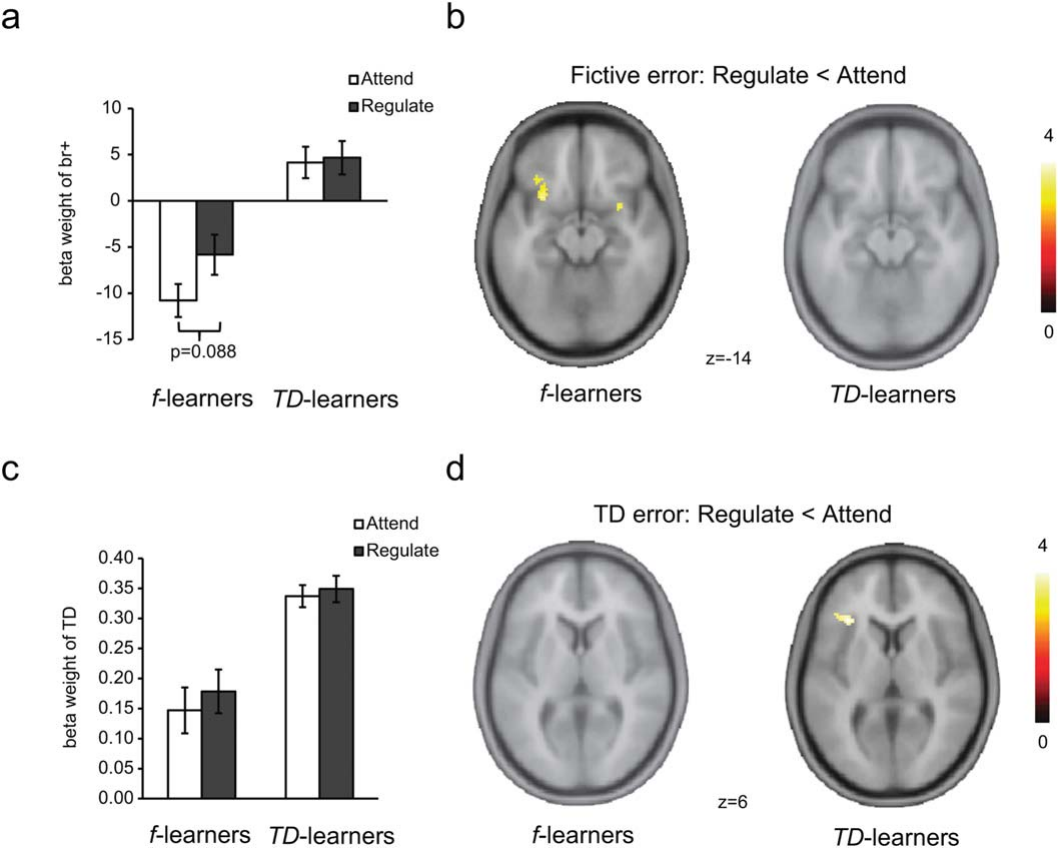
Learner type and task interaction. a) The modulatory effect of the reappraisal strategy on the weight of fictive gain *br*+ over the next bet was mostly driven by *f*-learners (*P* = 0.088) but not temporal different (TD)-learners (*P* > 0.8). b) The Regulate condition reduced fictive error related activation in left anterior insula in *f*-learners, but not TD-learners (*P* < 0.005 uncorrected, *k* > 10). c) The reappraisal strategy did not influence the weight of TD on next bet in either f-learners or TD-learners (*P*s > 0.2). d) The Regulate condition reduced TD error related activation in left anterior insula in TD-learners, but not *f*-learners (*P* < 0.005 uncorrected, *k* > 10). *br*+: interaction term of bet (*b*) and positive market return (*r*+). Error bars represent standard error.

We then explored the interaction between learner type and reappraisal (Fig. 5). Behaviorally, although the interaction between task and learner type was not significant (*P* > 0.2), planned comparison suggested a trend of reduced fictive learning in the Regulate condition in *f*-learners (*P* = 0.088), but not in TD-learners (*P* > 0.8; Fig. 5a). Regulate did not change the impact of TD on investment behavior in either group (Fig. 5c*; P*s > 0.2). The Regulate condition reduced fictive error-related AIC and OFC activations only in *f*-learners, but not in TD-learners (Fig. 5b and Supporting Information Table S13). The Regulate condition did not alter TD-related brain activation in *f*-learners; however, it reduced TD-related AIC and frontal activations in TD-learners (Fig. 5d and Supporting Information Table S14). Together with the behavioral findings, these results suggest that different learning strategies in *f*-learners and TD-learners are subserved by distinct neural correlates and such individual differences in learning interact with the reappraisal strategy.

## DISCUSSION

We provide human neuroimaging evidence demonstrating that fictive errors are more amenable to cognitive strategies such as reappraisal, when compared with reward prediction error signals; and that these learning signals and their interaction with cognitive influences vary among individual decision makers. These findings provide important insight into the dissociation in the nature of fictive and reward prediction error signals, the interaction between cognitive influences and these computational learning signals, and relevance to psychopathology and potential interventions.

### Reappraisal Strategy Selectively Interacts With Computational Learning Signals

The primary finding of the current study is the selective coupling between the reappraisal strategy and fictive error signals. In supervised actor-critic reinforcement learning [Rosenstein et al., 2004], TD errors derived from actually experienced rewards serve as the “critic” that is used to guide decision-making through the behavioral policies implemented by the “actor”. Errors derived from fictive outcomes (ongoing differences between what might have been achieved and what was actually achieved) have been considered as an important learning signal that complements classic TD errors, and hence, constitute a second type of “critic” [Chiu et al., 2008; Lohrenz et al., 2007]. It remained unclear how the “actor” balances between these two sources of critics. In the current study, change in cognitive contexts modulates the behavioral and neural correlates of fictive, but not reward prediction errors. We speculate that to implement such modulation, cognitive input selectively biases the weight of fictive errors on behavior through the “actor.” This also explains why fictive learning signals fail to guide decision-making in addicted individuals who lack behavioral control [Chiu et al., 2008]. An alternative account for the dissociation between TD learning and fictive learning would be that cognitive strategies modulate expected value signals, possibly at the level of the “actor.” This possibility could be supported by increased level of raw bets under the Regulate condition (see Results), where subjects' bets can serve as a proxy of expected value in the current paradigm. It is also consistent with previous finding of modulation of expected rewards of conditioned stimuli [Delgado et al., 2008].

Reward prediction errors, on the other hand, show robust resilience to the reappraisal strategy in the current study. This is in line with a previous finding that the impact of TD errors on behavior is not susceptible to addictive state and remains largely intact in chronic smokers [Chiu et al., 2008]. Although one previous study suggests that emotion regulation strategies can also modulate reward prediction errors [Staudinger et al., 2009], the strategy was different from our reappraisal strategy in that subjects were instructed to “distance” themselves from reward outcomes. Based on the selective coupling between the reappraisal strategy and fictive learning signals, we speculate that while both fictive and reward prediction errors guide decision-making, fictive signals might be more important in allowing behavioral flexibility while reward prediction errors are more robust and resilient to external modulatory factors. Such flexibility associated with fictive learning is important for developing potential intervention and treatment of psychopathology based on cognitive strategies.

### A role of the Anterior Insular Cortex and its Related Networks in Encoding Fictive Errors

In parallel with the behavioral findings, we find that the modulatory effect of cognitive strategies on fictive errors is primarily associated with reduced activity in the AIC and its associated brain regions such as the OFC and the striatum; as well as decreased AIC-amygdala connectivity. The AIC participates in a wide range of functions from low-level autonomic and interoceptive processes [Craig, 2009; Critchley et al., 2004], to high-level processes such as emotion [Fan et al., 2011; Lamm and Singer, 2010], empathy [Gu et al., 2010,^2012^,[Bibr b22]; Singer et al., 2004], fairness [Kirk et al., 2011; Sanfey et al., 2003], risk and uncertainty [Bossaerts, 2010; Preuschoff et al., 2008; Ullsperger et al., 2010], trust and cooperation [King-Casas et al., 2008], norm violations [Montague and Lohrenz, 2007; Xiang et al., 2013], and cognitive control [Eckert et al., 2009; Menon and Uddin, 2010]. Therefore, AIC is considered a critical neural substrate in integrating bodily signals with top-down control [Craig, 2009; Singer et al., 2009), potentially in a Bayesian optimal fashion [Gu et al., 2013a; Seth, 2013]. The amygdala is a critical structure in general emotional processing [Pessoa and Adolphs, 2010; Phelps, 2006] as well as Pavlovian learning [Li et al., 2011; Rangel et al., 2008]. Recent work has singled out a computational role of the amygdala in encoding economic uncertainty [Coricelli et al., 2005; De Martino et al., 2006; Hsu et al., 2005]. Importantly, a recent study suggests that economic risk-related amygdala activation in a gambling task is modulated by an emotion regulation strategy that is identical to the one used in the current study [Sokol-Hessner et al., 2013]. The AIC also has dense reciprocal connections with almost all subnuclei of the amygdaloid complex [Mufson et al., 1981], which provides the neuroanatomical basis for functional connectivity between these two structures. Both the AIC and amygdala are known to be involved in rapid information integration, especially when stimuli are salient and relevant [Eckert et al., 2009; Kuo et al., 2009; LeDoux, 2000].Therefore, we speculate that there are at least two aspects of the top-down modulatory effect on the activity and connectivity of the AIC in the current paradigm. Firstly, the AIC could compute the quantity of fictive errors per se; by selectively acting on AIC activity and connectivity, the reappraisal strategy is able to exert influence on the weight of fictive signals on behavior directly. Alternatively, the AIC could encode subjective feelings associated with fictive errors (e.g. regret); by modulating AIC activity and AIC-amygdala connectivity, the reappraisal strategy then modulates subjective feelings associated with fictive signals. These two aspects are possibly intertwined during economic decision-making both in our experimental setting as well as in real-life decision-making; and their dissociation remains to be examined by future studies.

### Individual Differences in Fictive and Reward Prediction Error Signals

In a post hoc analysis, we also identified individuals with different types of learning mechanisms, namely fictive learners and temporal difference learners; both types of learners have distinct behavioral and neural response patterns. Fictive learners' decisions are significantly driven by fictive outcomes and display robust neural activation related to fictive errors; the reappraisal strategy modulate these fictive learning signals only in *f*-learners. TD-learners' behaviors are under greater influence of TD errors and show TD-related activation in the striatum and OFC, compared with fictive learners; their neural responses to TD errors are also modulated by the reappraisal strategy although the behavioral modulatory effect is not significant. While these results should be interpreted with caution due to the post hoc nature of the analysis, they could contribute to the existing literature on different learning mechanisms among individuals, such as model-based vs. model-free learning [Daw et al., 2011; Glascher et al., 2010] by demonstrating individual differences in learning from fictive and reward prediction errors under the modified actor-critic model as proposed earlier in the Discussion and as elsewhere [Chiu et al., 2008; Lohrenz et al., 2007]. Under such framework, it is not surprising that individual decision-makers exhibit varying capacities to make decisions based on fictive rewards, and that the same cognitive strategies interact with individual behavioral and neural responses to fictive outcomes differently. These findings could be informative for cognitive intervention and treatment programs of psychopathology by suggesting that individual differences in decision-making and learning should be taken into account.

## CONCLUSION

Taken together, our results support the hypothesis that top-down cognitive strategies such as reappraisal can impact learning signals known to guide valuation and choice. Our findings are mainly twofold. First, while both fictive and reward prediction errors serve as important learning signals, only fictive signals are susceptible to cognitive strategies both behaviorally and neurally. Second, we present the first report of individual differences in fictive errors and its interaction with cognitive modulation. Overall**,** these findings suggest that the variable coupling of cognitive strategies to two important classes of learning signals (fictive, reward prediction error) represent one contributing substrate for the variable capacity of individuals to control their behavior based on foregone rewards. These findings also expose important possibilities for understanding the control or lack of control in addiction based on possibly foregone rewarding outcomes.
