# Supplementary material for: Cognitive Strategies Regulate Fictive, but not Reward Prediction Error Signals in a Sequential Investment Task
Source: Hum Brain Mapp. 2013 Dec 31;35(8):3738–49. doi: 10.1002/hbm.22433 (PMC4105325; doi:10.1002/hbm.22433)
Supplement: Supplementary file 1 — Supporting Information [file hbm0035-3738-SD1.docx]

**Cognitive Strategies Regulate Fictive, But Not Reward Prediction Error Signals in A Sequential Investment Task**

Xiaosi Gu, Ulrich Kirk, Terry M. Lohrenz, and P. Read Montague

**Supplementary Material**

**Instructions for participants**

*Attend.*

When you see Attend on the screen, focus on each of the following monetary decisions in complete isolation from all other decisions. Tell yourself it is the only choice that matters, that this one choice will determine whether you earn or lose money. As such, you might earn money, if the market goes up, but you could just as easily lose money, if the market goes down. Approach each decision as if you are making only this one choice in today’s study. Ask yourself how you would feel if you won money, how you would feel if you lost money. Just let any thoughts or emotions about that particular choice occur naturally, without trying to control them. It is important that you focus on the monetary decision in front of you at that time, in isolation from any context.

*Regulate.*

When you see Regulate on the screen, think of each of the following monetary decisions in the context of all of the previous and following choices. That is, treat it as one of many monetary decisions, which will constitute a ‘portfolio.’ Remind yourself that you are making many of these similar decisions. Do not keep a running total - simply approach these investment decisions keeping in mind their context.

Imagine you are considering one of the monetary decisions in this task right now.

One way to think of this instruction is to imagine yourself as a trader. You take risks with money every day, for a living. Imagine that this is your job and that the money at stake is not yours - it is someone else’s. Of course, you still want to do well (your job depends on it). You have done this for a long time, though, and will continue to. All that matters is that you come out on top in the end - a loss or gain here or there will not matter in terms of your overall portfolio. In other words, you win some and you lose some.

**Table S1** Fictive error *f ^+^* related activation: Attend (*P*<0.005 uncorrected, k>10)

| **Region** | **Laterality** | **x** | **y** | **z** | **Z** | **k** |
| --- | --- | --- | --- | --- | --- | --- |
| Superior occipital gyrus | R | 24 | -92 | 12 | 6.68 | 17383 |
| Cerebellum | L | -26 | -66 | -16 | 5.97 |  |
| Cerebellum | L | -30 | -52 | -24 | 5.45 |  |
| Midbrain |  | 8 | -24 | -12 | 4.52 |  |
| Caudate |  | -12 | 2 | 12 | 3.80 |  |
| Superior medial frontal gyrus | L | 2 | 54 | 20 | 4.98 | 313 |
| Superior medial frontal gyrus | R | 6 | 56 | 38 | 4 |  |
| Anterior cingulate |  | 0 | 42 | 16 | 3.96 |  |
| Orbitofrontal gyrus | L | -46 | 18 | -6 | 4.37 | 973 |
| Orbitofrontal gyrus | L | -34 | 24 | -14 | 4.19 |  |
| Insula | L | -32 | 14 | -10 | 4.03 |  |
| Orbitofrontal gyrus | R | 46 | 26 | -12 | 4.31 | 89 |
| Amygdala | R | 30 | 6 | -18 | 4.14 |  |
| Orbitofrontal gyrus | R | 30 | 18 | -20 | 3.67 |  |
| Superior parietal gyrus | R | 16 | -58 | 64 | 3.93 | 362 |
| Precuneus | R | 8 | -56 | 66 | 3.74 |  |
| Precuneus | L | -6 | -56 | 66 | 3.09 |  |
| Precentral gyrus | R | 36 | -16 | 50 | 3.78 | 725 |
| Precentral gyrus | R | 40 | -8 | 62 | 3.67 |  |
| Superior frontal gyrus | R | 22 | 0 | 70 | 3.35 |  |
| Superior parietal gyrus | L | -16 | -74 | 54 | 3.36 | 16 |
| Middle temporal gyrus | L | -62 | -16 | -14 | 3.32 | 13 |
| Precentral gyrus | R | 62 | 12 | 20 | 3.29 | 34 |
| Superior parietal gyrus | L | -22 | -56 | 58 | 3.09 | 52 |
| Postcentral gyrus | L | -26 | -46 | 56 | 2.75 |  |
| Precentral gyrus | L | -38 | -12 | 56 | 3.05 | 31 |
| Supplementary motor area | R | 6 | 0 | 52 | 2.9 | 19 |
| Precentral gyrus | L | -24 | -12 | 50 | 2.88 | 13 |

**Table S2** Fictive error *f ^+^* related activation: Regulate (*P*<0.005 uncorrected, k>10)

| **Region** | **Laterality** | **x** | **y** | **z** | **Z** | **k** |
| --- | --- | --- | --- | --- | --- | --- |
| Cuneus | R | 20 | -96 | 14 | 7.61 | 6011 |
| Lingual | L | -18 | -78 | -2 | 4.88 |  |
| Lingual | L | -8 | -76 | -4 | 4.8 |  |
| Superior frontal gyrus | L | -28 | -4 | 66 | 3.92 | 637 |
| Superior frontal gyrus | L | -24 | -4 | 58 | 3.52 |  |
| Superior frontal gyrus | L | -20 | 4 | 60 | 3.2 |  |
| Superior frontal gyrus | R | 22 | -10 | 72 | 3.67 | 861 |
| Superior frontal gyrus | R | 24 | -2 | 70 | 3.54 |  |
| Precentral gyrus | R | 36 | -20 | 54 | 3.47 |  |
| Cerebellum |  | 0 | -62 | -36 | 3.63 | 272 |
| Cerebellum | L | -6 | -52 | -36 | 3.38 |  |
| Cerebellum | R | 10 | -60 | -34 | 2.85 |  |
| Precuneus | L | -14 | -62 | 64 | 3.53 | 132 |
| Precuneus | L | -12 | -56 | 54 | 2.72 |  |
| Supplementary motor area | R | 10 | -2 | 54 | 3.49 | 87 |
| Supplementary motor area | R | 18 | -8 | 54 | 2.75 |  |
| Supplementary motor area |  | 0 | 4 | 70 | 3.3 | 80 |
| Rectus | L | -4 | 30 | -18 | 3.1 | 70 |
| Orbitofrontal gyrus |  | 0 | 40 | -8 | 3.04 |  |
| Cerebellum | L | -18 | -54 | -50 | 3.09 | 36 |
| Rectus | L | -10 | 24 | -10 | 3.07 | 20 |
| Precentral gyrus | L | -60 | 2 | 30 | 3.07 | 10 |
| Superior parietal gyrus | R | 20 | -60 | 60 | 2.95 | 13 |
| Postcentral gyrus | L | -54 | -2 | 42 | 2.92 | 15 |
| Cerebellum |  | 4 | -52 | 2 | 2.84 | 12 |
| Postcentral gyrus | L | -42 | -28 | 48 | 2.84 | 17 |
| Precuneus | R | 4 | -54 | 64 | 2.76 | 24 |

**Table S3** Reappraisal effect on *f ^+^*: Regulate < Attend (*P*<0.005 uncorrected, k>10)

| **Region** | **Laterality** | **x** | **y** | **z** | **Z** | **k** |
| --- | --- | --- | --- | --- | --- | --- |
| Inferior frontal gyrus | R | 50 | 32 | 8 | 3.9 | 273 |
| Inferior frontal gyrus | R | 56 | 22 | 10 | 2.96 |  |
| Orbitofrontal gyrus | R | 48 | 30 | -8 | 2.69 |  |
| Supramarginal gyrus | R | 62 | -42 | 44 | 3.49 | 88 |
| Supramarginal gyrus | R | 64 | -30 | 32 | 2.7 |  |
| Middle temporal gyrus | L | -64 | -34 | -8 | 3.37 | 222 |
| Middle temporal gyrus | L | -62 | -16 | -14 | 3.21 |  |
| Middle temporal gyrus | L | -50 | -32 | -8 | 3.02 |  |
| Orbitofrontal gyrus | L | -36 | 30 | -12 | 3.35 | 58 |
| Thalamus | L | -22 | -22 | 0 | 3.3 | 29 |
| Insula (extending into striatum) | R | -34 | 8 | 0 | 3.27 | 39 |
| Angular | R | 56 | -62 | 32 | 3.27 | 92 |
| Angular | R | 48 | -58 | 30 | 3.01 |  |
| Angular | R | 60 | -56 | 36 | 2.69 |  |
| Middle temporal gyrus | R | 64 | -30 | -6 | 3.18 | 122 |
| Superior medial frontal gyrus | L | -2 | 52 | 16 | 3.13 | 145 |
| Parahippocampal gyrus | R | 24 | -36 | -4 | 3.12 | 12 |
| Middle temporal gyrus | L | -64 | -50 | 2 | 3.1 | 26 |
| Thalamus | L | -16 | -30 | 6 | 3.08 | 35 |
| Hippocampal gyrus | R | 28 | -20 | -16 | 3.03 | 36 |
| Substantia nigra | R | 14 | -16 | -14 | 2.98 | 18 |
| Posterior cingulate | L | -4 | -50 | 26 | 2.97 | 79 |
| Hippocampal gyrus | R | 6 | -50 | 24 | 2.93 |  |
| Middle temporal gyrus | L | -44 | -58 | 24 | 2.97 | 57 |
| Inferior frontal gyrus | L | -46 | 28 | 2 | 2.83 | 18 |
| Hippocampal gyrus | L | -18 | -12 | -18 | 2.83 | 10 |
| Superior medial frontal gyrus | R | 10 | 52 | 40 | 2.82 | 32 |
| Superior medial frontal gyrus | L | 2 | 46 | 40 | 2.76 |  |
| Cerebellum | L | -24 | -66 | -34 | 2.77 | 18 |

**Table S4** Fictive error *f ^+^* related activation (averaged across Attend and Regulate; *P*<0.005 uncorrected, k>10)

| **Region** | **Laterality** | **x** | **y** | **z** | **Z** | **k** |
| --- | --- | --- | --- | --- | --- | --- |
| Superior occipital gyrus | R | 22 | -96 | 12 | Inf | 19034 |
| Cerebellum | L | -30 | -66 | -18 | 6.43 |  |
| Superior occipital gyrus | L | -10 | -96 | 4 | 6.16 |  |
| Caudate | L | -16 | -4 | 14 | 3.89 |  |
| Pallidum | R | 24 | -14 | -4 | 3.77 |  |
| Precuneus | R | 6 | -54 | 66 | 4.18 | 881 |
| Superior parietal gyrus | R | 18 | -60 | 60 | 3.79 |  |
| Superior parietal gyrus | L | -20 | -60 | 60 | 3.74 |  |
| Superior frontal gyrus | R | 24 | -2 | 70 | 4.09 | 2899 |
| Precentral gyrus | R | 34 | -16 | 50 | 4 |  |
| Superior frontal gyrus | R | 22 | -10 | 72 | 3.95 |  |
| Insula | L | -34 | 16 | -12 | 3.65 | 322 |
| Insula | L | -44 | 16 | -6 | 3.61 |  |
| Superior temporal gyrus | L | -44 | 18 | -22 | 3.46 |  |
| Orbitofrontal gyrus | R | 42 | 22 | -10 | 3.59 | 285 |
| Orbitofrontal gyrus | R | 30 | 16 | -20 | 3.58 |  |
| Orbitofrontal gyrus | R | 24 | 30 | -18 | 3.57 | 42 |
| Precentral gyrus | R | 60 | 10 | 24 | 3.46 | 61 |
| Precentral gyrus | L | -60 | 2 | 30 | 3.22 | 64 |
| Precentral gyrus | L | -56 | 0 | 38 | 3.02 |  |
| Superior medial frontal gyrus | R | 8 | 56 | 36 | 3.11 | 19 |
| Postcentral gyrus | L | -42 | -28 | 46 | 2.82 | 22 |
| Cerebelum | R | 34 | -56 | -48 | 2.77 | 13 |

**Table S5** Psychophysiological interaction analysis reveals decreased functional connectivity under Regulate compared to Attend *(P*<0.005 uncorrected, k>10)

| **Region** | **Lateraility** | **x** | **y** | **z** | **Z** | **k** |
| --- | --- | --- | --- | --- | --- | --- |
| Middle temporal gyrus | R | 68 | -40 | -2 | 3.75 | 231 |
| Middle temporal gyrus | R | 68 | -32 | 2 | 3.58 |  |
| Middle temporal gyrus | R | 62 | -32 | -10 | 3.46 |  |
| Heschl gyrus | R | 38 | -22 | 16 | 3.46 | 81 |
| Amygdala | R | 32 | -4 | -14 | 3.28 | 115 |
| Hippocampus | R | 28 | -10 | -18 | 2.99 |  |
| Hippocampus | R | 20 | -8 | -18 | 2.96 |  |
| Middle temporal gyrus | R | 56 | -52 | 22 | 3.17 | 68 |
| Superior temporal gyrus | L | -48 | -20 | 14 | 3.16 | 91 |
| Rolandic operculum | R | 52 | -12 | 12 | 3 | 19 |
| Olfactory | R | 4 | 12 | -8 | 2.9 | 12 |

**Table S6** Temporal difference (*TD)* error related activation: Attend (*P*<0.05 corrected for family wise error and k>5)

| **Region** | **Laterality** | **x** | **y** | **z** | **Z** | **k** |
| --- | --- | --- | --- | --- | --- | --- |
| Putamen | L | -14 | 10 | -8 | 7.77 | 4452 |
| Putamen | R | 16 | 10 | -10 | 7.52 |  |
| Orbitofrontal gyrus | L | -32 | 54 | -2 | 7.24 |  |
| Middle frontal gyrus | L | -26 | 18 | 52 | 6.27 | 1160 |
| Middle frontal gyrus | L | -32 | 28 | 40 | 5.28 |  |
| Middle frontal gyrus | L | -44 | 20 | 38 | 5.19 |  |
| Inferior parietal gyrus | L | -48 | -44 | 40 | 6.17 | 840 |
| Inferior parietal gyrus | L | -48 | -54 | 46 | 5.68 |  |
| Inferior parietal gyrus | L | -42 | -56 | 52 | 5.58 |  |
| Precuneus | L | -10 | -58 | 58 | 6.01 | 5105 |
| Paracentral lobule | R | 10 | -40 | 60 | 5.95 |  |
| Superior occipital gyrus | R | 22 | -68 | 42 | 5.81 |  |
| Inferior temporal gyrus | L | -52 | -44 | -12 | 5.61 | 223 |
| Middle temporal gyrus | L | -64 | -28 | -6 | 5.38 |  |
| Middle temporal gyrus | L | -56 | -32 | -10 | 5.07 |  |
| Superior medial frontal gyrus | L | -6 | 26 | 44 | 5.53 | 208 |
| Anterior cingulate | L | -8 | 38 | 26 | 4.82 |  |
| Frontoinsula | R | 38 | -32 | 30 | 5.37 | 193 |
| Frontoinsula | R | 36 | -22 | 34 | 4.98 |  |
| Frontoinsula | R | 44 | -38 | 28 | 4.68 |  |
| Orbitofrontal gyrus | R | 40 | 46 | -10 | 5.34 | 30 |
| Superior temporal gyrus | R | 62 | -6 | -4 | 5.27 | 108 |
| Superior temporal gyrus | R | 60 | 2 | -4 | 5 |  |
| Middle temporal gyrus | R | 66 | -14 | -12 | 4.57 |  |
| Cerebellum | R | 32 | -66 | -38 | 5.22 | 180 |
| Middle temporal gyrus | L | -42 | -60 | 10 | 5.14 | 24 |
| Precentral gyrus | L | -46 | -4 | 46 | 5.01 | 68 |
| Inferior temporal gyrus | L | -56 | -58 | -4 | 4.92 | 39 |
| Precentral gyrus | L | -32 | -16 | 46 | 4.88 | 40 |
| Precentral gyrus | L | -32 | -4 | 46 | 4.79 |  |
| Precentral gyrus | L | -52 | 4 | 18 | 4.88 | 32 |
| Superior frontal gyrus | R | 26 | 24 | 50 | 4.87 | 62 |
| Rectus | R | 6 | 30 | -18 | 4.84 | 25 |
| Rectus | L | -6 | 32 | -16 | 4.71 |  |
| Precuneus | R | 24 | -60 | 22 | 4.82 | 20 |
| Inferior parietal gyrus | R | 50 | -50 | 50 | 4.81 | 70 |
| Supramarginal gyrus | R | 56 | -44 | 46 | 4.69 |  |
| Inferior parietal gyrus | R | 60 | -48 | 40 | 4.63 |  |
| Precuneus | R | 10 | -62 | 56 | 4.77 | 23 |
| Superior frontal gyrus | L | -24 | -8 | 64 | 4.75 | 9 |
| Superior temporal gyrus | R | 62 | -32 | 6 | 4.74 | 31 |
| Supramarginal gyrus | R | 50 | -32 | 38 | 4.68 | 7 |
| Middle occipital gyrus | L | -20 | -84 | 18 | 4.65 | 6 |

**Table S7** Temporal difference (*TD)* error - related activation: Regulate (*P*<0.05 corrected for family wise error and k>5)

| **Region** | **Laterality** | **x** | **y** | **z** | **Z** | **k** |
| --- | --- | --- | --- | --- | --- | --- |
| Putamen | L | -16 | 8 | -10 | Inf | 5494 |
| Putamen | R | 10 | 6 | -8 | 7.75 |  |
| Putamen | R | 18 | 10 | -8 | 7.69 |  |
| Precuneus | R | 16 | -52 | 34 | 6.38 | 635 |
| Precuneus | L | 18 | -40 | 30 | 5.28 |  |
| Precuneus | L | 16 | -48 | 26 | 4.91 |  |
| Cerebellum | R | 42 | -64 | -36 | 6.11 | 167 |
| Middle occipital gyrus | R | 30 | -92 | 12 | 6 | 202 |
| Precuneus | R | 12 | -46 | 70 | 5.75 | 476 |
| Paracentral lobule | R | 6 | -42 | 64 | 5.19 |  |
| Paracentral lobule | L | -6 | -26 | 74 | 5.13 |  |
| Fusiform | R | 42 | -18 | -18 | 5.61 | 59 |
| Superior frontal gyrus | L | -24 | 54 | 4 | 5.27 | 408 |
| Middle frontal gyrus | L | -32 | 6 | 14 | 5.7 |  |
| Middle frontal gyrus | L | -30 | 52 | -6 | 4.77 |  |
| Middle frontal gyrus | R | 34 | 28 | 48 | 5.13 | 152 |
| Middle frontal gyrus | R | 30 | 38 | 44 | 4.85 |  |
| Middle temporal gyrus | L | -62 | -16 | -2 | 4.86 | 52 |
| Middle temporal gyrus | L | -62 | -34 | -12 | 4.81 |  |
| Middle temporal gyrus | L | -64 | -24 | -2 | 4.65 |  |
| Insula | L | -30 | 24 | 14 | 4.73 | 12 |
| Middle frontal gyrus | R | 30 | 52 | 8 | 4.72 | 38 |
| Superior frontal gyrus | R | 18 | 54 | 6 | 4.62 |  |
| Superior parietal gyrus | R | 14 | -7 | 56 | 4.72 | 22 |
| Paracentral lobule | L | -10 | -16 | 72 | 4.68 | 17 |
| Superior occipital gyrus | R | 30 | -76 | 44 | 4.66 | 11 |
| Inferior frontal gyrus | L | -38 | 36 | 12 | 4.66 | 7 |
| Middle temporal gyrus | R | 62 | -26 | -12 | 4.65 | 9 |
| Precentral gyrus | L | -24 | -22 | 72 | 4.62 | 6 |
| Superior temporal gyrus | L | -52 | -26 | 8 | 4.61 | 8 |
| Orbitofrontal gyrus | L | -42 | 44 | -2 | 4.59 | 7 |
| Superior medial frontal gyrus | R | 8 | 26 | 44 | 4.56 | 8 |

**Table S8** Reappraisal effect on temporal difference (*TD)* error: Regulate > Attend *(P*<0.005 uncorrected, k>10)

| **Region** | **Laterality** | **x** | **y** | **z** | **Z** | **k** |
| --- | --- | --- | --- | --- | --- | --- |
| Fusiform gyrus | L | -4 | -44 | -6 | 3.98 | 653 |
| Hippocampus | L | -36 | -3 | -12 | 3.96 |  |
| Hippocampus | R | 36 | -14 | -2 | 3.92 | 125 |
| Fusiform gyrus | R | 34 | -46 | 6 | 3.25 | 79 |
| Calcarine | R | 2 | -46 | 1 | 3.18 |  |
| Fusiform gyrus | R | 36 | -36 | 2 | 2.69 |  |
| Fusiform gyrus | L | -36 | 0 | -22 | 3.13 | 35 |
| Substantia nigra | R | 6 | -1 | -12 | 3.2 | 5 |
| Cerebellum | L | -1 | -36 | -18 | 2.89 | 15 |

**Table S9** Brain activations related to fictive error *f ^+^* by learner type *(P*<0.005 uncorrected, k>10). *TD*, temporal difference.

| **Region** | **Laterality** | **x** | **y** | **z** | **Z** | **k** |
| --- | --- | --- | --- | --- | --- | --- |
| ***f-learners*** |  |  |  |  |  |  |
| Superior occipital gyrus | R | 22 | -96 | 12 | 6.94 | 23466 |
| Cerebellum | L | -28 | -66 | -16 | 5.5 |  |
| Lingual gyrus | R | 4 | -72 | 4 | 5.28 |  |
| Orbitofrontal gyrus | L | -34 | 2 | -14 | 4.51 | 617 |
| Insula | L | -24 | 16 | -16 | 3.74 |  |
| Insula | L | -42 | 16 | -4 | 3.68 |  |
| Precuneus | R | 2 | -54 | 6 | 4.27 | 2427 |
| Superior parietal gyrus | R | 18 | -56 | 64 | 4.21 |  |
| Superior parietal gyrus | L | -16 | -6 | 58 | 4.1 |  |
| Cerebellum | L | -20 | -52 | -5 | 3.63 | 56 |
| Hippocampus | L | -24 | -24 | -8 | 3.55 | 27 |
| Hippocampus | L | -26 | -32 | -2 | 3.41 |  |
| Precentral | L | -52 |  | 38 | 3.34 | 69 |
| Superior medial frontal gyrus | R | 14 | 58 | 34 | 3.29 | 4 |
| Postcentral gyrus | L | -42 | -36 | 34 | 3.9 | 149 |
| Postcentral gyrus | L | -40 | -34 | 46 | 2.78 |  |
| Superior occipital gyrus | R | 30 | -78 | 4 | 2.91 | 14 |
| Posterior cingulate | L | 0 | -22 | 34 | 2.81 | 34 |
| Posterior cingulate | L | -4 | -14 | 34 | 2.67 |  |
|  |  |  |  |  |  |  |
| ***TD-learners*** |  |  |  |  |  |  |
| Cuneus | R | 20 | -96 | 12 | 7.26 | 1312 |
| Fusiform | R | 34 | -58 | -18 | 4.14 |  |
| Fusiform | R | 38 | -70 | -12 | 3.44 |  |
| Cerebellum | L | -30 | -66 | -18 | 5.19 | 3163 |
| Superior occipital gyrus | L | -10 | -94 | 4 | 4.34 |  |
| Inferior occipital gyrus | L | -24 | -84 | -2 | 4.22 |  |
| Superior frontal gyrus | R | 22 | -8 | 72 | 3.79 | 50 |
| Precentral gyrus | R | 32 | -16 | 48 | 3.37 | 245 |
| Precentral gyrus | R | 42 | -14 | 62 | 3.12 |  |
| Precentral gyrus | R | 36 | -22 | 66 | 3.12 |  |
| Precentral gyrus | R | 62 | 6 | 28 | 2.77 | 12 |
| Postcentral gyrus | R | 54 | -14 | 44 | 2.72 | 11 |

**Table S10** Brain activations related to fictive error *f ^+^* : *f*-learners > *TD*-learners. No activation was found for *TD*-learners>*f*-learners *(P*<0.005 uncorrected, k>10). *TD*, temporal difference.

| **Region** | **Laterality** | **x** | **y** | **z** | **Z** | **k** |
| --- | --- | --- | --- | --- | --- | --- |
| ***f-learners > TD-learner*** |  |  |  |  |  |  |
| Precuneus | R | 8 | -48 | 44 | 3.65 | 1227 |
| Midcingulate | R | 4 | -38 | 40 | 3.52 |  |
| Insula | R | 44 | 26 | -4 | 3.55 | 129 |
| Inferior temporal gyrus | R | 48 | -46 | -10 | 3.49 | 73 |
| Calcarine | L | -10 | -46 | 6 | 3.42 | 153 |
| Calcarine | L | -14 | -52 | 12 | 2.78 |  |
| Orbitofrontal gyrux | L | -8 | 42 | -10 | 3.39 | 124 |
| Anterior cingulate | L | -8 | 44 | -2 | 2.72 |  |
| Orbitofrontal gyrus | R | 12 | 42 | 0 | 3.37 | 176 |
| Rectus | R | 10 | 42 | -14 | 3.01 |  |
| Orbitofrontal gyrus | L | -34 | 24 | -16 | 3.35 | 137 |
| Orbitofrontal gyrus | L | -26 | 24 | -14 | 3.24 |  |
| Lingual | R | 12 | -50 | -2 | 3.34 | 341 |
| Precuneus | R | 8 | -46 | 14 | 3.08 |  |
| Calcarine | R | 20 | -52 | 6 | 2.94 |  |
| Inferior temporal gyrus | L | -44 | -54 | -10 | 3.29 | 65 |
| Middle frontal gyrus | L | -24 | 16 | 62 | 3.27 | 82 |
| Superior frontal gyrus | L | -16 | 14 | 62 | 2.94 |  |
| Anterior cingulate | R | 12 | 18 | 22 | 3.14 | 67 |
| Anterior cingulate | R | 14 | 28 | 30 | 3.08 |  |
| Superior occipital gyrus | L | -24 | -76 | 16 | 3.07 | 44 |
| Superior occipital gyrus | L | -20 | -76 | 24 | 2.81 |  |
| Calcarine | R | 6 | -92 | 8 | 3.04 | 40 |
| Calcarine | L | 2 | -88 | 0 | 2.87 |  |
| Supramarginal gyrus | L | -46 | -38 | 32 | 3 | 12 |
| Fusiform | R | 22 | -38 | -12 | 2.98 | 51 |
| Fusiform | R | 34 | -44 | -6 | 2.97 |  |
| Parahippocampal gyrus | R | 32 | -36 | -10 | 2.77 |  |
| Thalamus |  | 0 | -28 | 4 | 2.94 | 94 |
| Thalamus | L | -8 | -20 | 12 | 2.79 |  |
| Lingual | R | 4 | -72 | 4 | 2.93 | 58 |
| Superior frontal gyrus | R | 18 | 56 | 36 | 2.89 | 16 |
| Calcarine | L | -4 | -62 | 14 | 2.82 | 32 |
| Cuneus | L | -14 | -66 | 22 | 2.73 |  |
| Middle occipital gyrus | L | -38 | -80 | 26 | 2.82 | 21 |
| Middle frontal gyrus | L | -24 | 34 | 30 | 2.79 | 28 |
| Middle frontal gyrus | L | -24 | 34 | 30 | 2.79 | 28 |

**Table S11** Brain activations related to temporal difference (*TD)* error by learner type (*P*<0.05 corrected for family wise error and k>5)

| **Region** | **Laterality** | **x** | **y** | **z** | **Z** | **k** |
| --- | --- | --- | --- | --- | --- | --- |
| ***f-learners*** |  |  |  |  |  |  |
| Putamen | R | 8 | 6 | -8 | 6.15 | 1052 |
| Putamen | L | -14 | 10 | -8 | 5.98 |  |
| Putamen | R | 16 | 10 | -10 | 5.91 |  |
| Precuneus | R | 10 | -42 | 62 | 5.53 | 426 |
| Precentral | R | 20 | -28 | 72 | 5.04 |  |
| Superior parietal gyrus | R | 18 | -56 | 62 | 4.8 |  |
| Orbitofrontal gyrus | L | -34 | 52 | -6 | 5.06 | 140 |
| Middle frontal gyrus | L | -32 | 46 | 0 | 5.05 |  |
| Inferior temporal gyrus | L | -48 | -22 | -18 | 4.99 | 50 |
| Putamen | L | -30 | -6 | -4 | 4.94 | 72 |
| Middle temporal gyrus | L | -52 | -36 | -6 | 4.81 | 36 |
| Paracentral lobule | L | -8 | -28 | 72 | 4.78 | 24 |
| Middle frontal gyrus | L | -24 | 20 | 50 | 4.77 | 21 |
| Middle temporal gyrus | L | -58 | -16 | -8 | 4.75 | 14 |
| Orbitofrontal gyrus | L | -44 | 40 | -8 | 4.69 | 9 |
| Paracentral lobule | L | -6 | -16 | 66 | 4.63 | 10 |
|  |  |  |  |  |  |  |
| ***TD-learners*** |  |  |  |  |  |  |
| Putamen | L | -14 | 10 | -8 | 6.71 | 2002 |
| Caudate | R | 14 | 10 | -8 | 6.53 |  |
| Superior occipital gyrus | R | 22 | -88 | 20 | 6.6 | 2550 |
| Middle occipital gyrus | R | 28 | -90 | 12 | 6.19 |  |
| Superior occipital gyrus | R | 22 | -84 | 32 | 6 |  |
| Superior medial frontal gyrus | R | 8 | 34 | 46 | 5.76 | 322 |
| Superior medial frontal gyrus | L | -10 | 36 | 30 | 4.83 |  |
| Superior medial frontal gyrus | L | -8 | 30 | 42 | 4.77 |  |
| Orbitofrontal gyrus | L | -30 | 54 | -2 | 5.61 | 836 |
| Orbitofrontal gyrus | L | -36 | 48 | 0 | 5.5 |  |
| Inferior frontal gyrus | L | -38 | 42 | 6 | 5.02 |  |
| Cerebellum | R | 42 | -66 | -36 | 5.58 | 242 |
| Orbitofrontal gyrus | R | 26 | 54 | -2 | 5.36 | 206 |
| Superior frontal gyrus | R | 20 | 54 | 10 | 4.94 |  |
| Middle frontal gyrus | R | 30 | 52 | 8 | 4.58 |  |
| Middle frontal gyrus | R | 36 | 24 | 48 | 5.34 | 223 |
| Middle frontal gyrus | R | 46 | 24 | 44 | 5.23 |  |
| Inferior parietal gyrus | L | -48 | -54 | 46 | 5.24 | 125 |
| Middle temporal gyrus | L | -56 | -34 | -8 | 5.12 | 76 |
| Middle temporal gyrus | L | -64 | -28 | -8 | 4.71 |  |
| Rectus | L | -4 | 32 | -14 | 5.12 | 179 |
| Paracentral lobule | L | -6 | -22 | 76 | 4.99 | 25 |
| Paracentral lobule | L | -8 | -14 | 76 | 4.9 |  |
| Superior occipital gyrus | L | -18 | -68 | 24 | 4.98 | 414 |
| Middle occipital gyrus | L | -20 | -82 | 18 | 4.88 |  |
| Middle frontal gyrus | L | -26 | 20 | 38 | 4.89 | 61 |
| Orbitofrontal gyrus | L | -26 | 26 | -14 | 4.87 | 10 |
| Middle frontal gyrus | L | -38 | 24 | 40 | 4.81 | 77 |
| Lingual gyrus | R | 12 | -76 | -2 | 4.73 | 28 |
| Middle occipital gyrus | L | -38 | -68 | 4 | 4.7 | 9 |
| Precuneus | R | 12 | -46 | 70 | 4.69 | 8 |
| Frontal operculum | R | 32 | 14 | 30 | 4.68 | 10 |
| Middle temporal gyrus | R | 62 | -24 | -8 | 4.6 | 11 |
| Cuneus |  | 0 | -76 | 36 | 4.58 | 12 |

**Table S12** Brain activations related to TD: *TD*-learners>*f*-learners. No activation was found for *f*-learners>*TD*-learners *(P*<0.005 uncorrected, k>10). *TD*, temporal difference.

| **Region** | **Laterality** | **x** | **y** | **z** | **Z** | **k** |
| --- | --- | --- | --- | --- | --- | --- |
| ***TD-learners > f-learner*** |  |  |  |  |  |  |
| Superior occipital gyrus | R | 18 | -88 | 22 | 3.91 | 1940 |
| Lingual | R | 12 | -78 | -4 | 3.64 |  |
| Cuneus | R | 8 | -88 | 24 | 3.2 |  |
| Orbitofrontal gyrus | R | 28 | 54 | -4 | 3.21 | 69 |
| Fusiform | R | 28 | -76 | -8 | 2.94 | 51 |
| Inferior occipital gyrus | R | 30 | -82 | -16 | 2.71 |  |
| Cerebellum | R | 34 | -68 | -18 | 2.94 | 39 |
| Orbitofrontal gyrus | R | 2 | 58 | -12 | 2.82 | 15 |

**Table S13** Task difference in brain activations related to fictive error *f ^+^*. No activation was found for *TD*-learners>*f*-learners. *TD*, temporal difference. *(P*<0.005 uncorrected, k>10; **P*<0.05 uncorrected for regions of interest).

| **Region** | **Laterality** | **x** | **y** | **z** | **Z** | **k** |
| --- | --- | --- | --- | --- | --- | --- |
| ***f-learners: Regulate < Attend*** | | | | | | |
| Cerebellum | L | -24 | -64 | -42 | 3.73 | 229 |
| Cerebellum | L | -26 | -42 | -34 | 3.58 |  |
| Cerebellum | L | -10 | -32 | -34 | 3.12 |  |
| Middle temporal gyrus | R | 64 | -32 | -12 | 3.38 | 36 |
| Insula | L | -30 | 14 | -14 | 3.33 | 40 |
| Inferior frontal gyrus | R | 48 | 32 | 10 | 3.16 | 50 |
| Angular | L | -46 | -60 | 28 | 3.03 | 38 |
| Amygdala | R | 30 | 4 | -18 | 3.02 | 30 |
| Orbitofrontal gyrus | L | -36 | 30 | -12 | 2.92 | 16 |
| Inferior frontal gyrus | L | -44 | 26 | 2 | 2.9 | 33 |
| Inferior frontal gyrus | L | -54 | 30 | 2 | 2.67 |  |
| SupraMarginal | R | 62 | -42 | 44 | 2.86 | 10 |
| SupraMarginal | R | 66 | -38 | 36 | 2.78 |  |
| Angular | R | 54 | -58 | 32 | 2.8 | 15 |
|  |  |  |  |  |  |  |
| ***TD-learners: Regulate < Attend*** |  |  |  |  |  |  |
| Supramarginal gyrus | R | 62 | -30 | 28 | 2.87 | 15 |
| Superior medial prefrontal gyrus | L | 0 | 54 | 18 | 2.87 | 100 |
| Superior medial prefrontal gyrus | R | 8 | 52 | 28 | 2.72 |  |
| Middle temporal gyrus | L | -50 | -28 | -10 | 2.68 | 14 |
|  |  |  |  |  |  |  |
| ***f-learners > TD-learners: Regulate < Attend*** | | | | | | |
| Cerebellum | L | -8 | -60 | 2.73 | 2.63 | 380 |
| Insula* | R | 34 | 22 | 2.2 | 2.14 | 22 |
| Orbitofrontal gyrus* | R | 20 | 48 | 2.09 | 2.04 | 17 |
| Insula* | L | -30 | 14 | 2.04 | 2 | 17 |

**Table S14** Task difference in brain activations related to *TD* (temporal difference). No activation was found for *f*-learners>*TD*-learners. *(P*<0.005 uncorrected, k>10; **P*<0.05 uncorrected for regions of interest).

| **Region** | **Laterality** | **x** | **y** | **z** | **Z** | **k** |
| --- | --- | --- | --- | --- | --- | --- |
| ***f-learners: Regulate < Attend*** |  |  |  |  |  |  |
| None |  |  |  |  |  |  |
|  |  |  |  |  |  |  |
| ***TD-learners: Regulate < Attend*** | | | | | | |
| Insula | L | -34 | 28 | 6 | 3.2 | 80 |
| Inferior frontal gyrus | L | -44 | 32 | 4 | 2.66 |  |
| Orbitofrontal gyrus | L | -28 | 28 | -12 | 2.94 | 28 |
| Insula | L | -26 | 26 | -4 | 2.69 |  |
| Cerebellum | R | 28 | -76 | -38 | 2.91 | 32 |
| Cerebellum | R | 18 | -76 | -44 | 2.7 |  |
|  |  |  |  |  |  |  |
| ***TD-leaners > f-learners: Regulate < Attend*** | | | | | | |
| Middle frontal gyrus | L | -36 | 48 | 28 | 3.34 | 35 |
| Cerebellum | L | -16 | -78 | -40 | 3.16 | 92 |
| Cerebellum | L | -22 | -74 | -44 | 2.99 |  |
| Vermis | R | 2 | -72 | -24 | 2.94 | 68 |
| Cerebellum | R | 10 | -76 | -18 | 2.76 |  |
| Superior frontal gyrus | R | 32 | -8 | 66 | 2.93 | 12 |
| Cerebellum | R | 10 | -66 | -14 | 2.89 | 17 |
| Insula* | L | -38 | 30 | 8 | 2.05 |  |
